# Scribble co-operatively binds multiple α_1D_-adrenergic receptor C-terminal PDZ ligands

**DOI:** 10.1038/s41598-019-50671-6

**Published:** 2019-10-01

**Authors:** Eric M. Janezic, Dorathy-Ann Harris, Diana Dinh, Kyung-Soon Lee, Aaron Stewart, Thomas R. Hinds, Peter L. Hsu, Ning Zheng, Chris Hague

**Affiliations:** 1 Department of Pharmacology, School of Medicine, University of Washington, 1959 NE Pacific Street, Seattle, WA 98195 USA; 2 Howard Hughes Medical Institute, University of Washington, Seattle, WA 98195 USA

**Keywords:** Supramolecular assembly, G protein-coupled receptors

## Abstract

Many G protein-coupled receptors (GPCRs) are organized as dynamic macromolecular complexes in human cells. Unraveling the structural determinants of unique GPCR complexes may identify unique protein:protein interfaces to be exploited for drug development. We previously reported α_1D_-adrenergic receptors (α_1D_-ARs) – key regulators of cardiovascular and central nervous system function – form homodimeric, modular PDZ protein complexes with cell-type specificity. Towards mapping α_1D_-AR complex architecture, biolayer interferometry (BLI) revealed the α_1D_-AR C-terminal PDZ ligand selectively binds the PDZ protein scribble (SCRIB) with >8x higher affinity than known interactors syntrophin, CASK and DLG1. Complementary *in situ* and *in vitro* assays revealed SCRIB PDZ domains 1 and 4 to be high affinity α_1D_-AR PDZ ligand interaction sites. SNAP-GST pull-down assays demonstrate SCRIB binds multiple α_1D_-AR PDZ ligands via a co-operative mechanism. Structure-function analyses pinpoint R1110^PDZ4^ as a unique, critical residue dictating SCRIB:α_1D_-AR binding specificity. The crystal structure of SCRIB PDZ4 R1110G predicts spatial shifts in the SCRIB PDZ4 carboxylate binding loop dictate α_1D_-AR binding specificity. Thus, the findings herein identify SCRIB PDZ domains 1 and 4 as high affinity α_1D_-AR interaction sites, and potential drug targets to treat diseases associated with aberrant α_1D_-AR signaling.

## Introduction

G protein-coupled receptors (GPCRs) account for ~4% of the human genome and are targets for ~30% of FDA approved drugs^1^. Typically these medications compete with endogenous ligands for orthosteric binding sites, hindering drug selectivity due to the similarity of binding pockets amongst closely related GPCRs. Thus, there is great interest in identifying novel sites to modulate GPCR signaling. To this end, a growing body of research has focused on identifying and characterizing the functional roles of GPCR interacting proteins. Two prominent examples are the β-arrestins^2^; and PDZ (PSD95/Dlg/ZO-1) domain containing proteins, which typically interact with C-terminal PDZ ligands^3,4^. Since the discovery that rhodopsin interacts with inaD^5^ and β_2_-adrenergic receptor (AR) with NHERF^6^, significant effort has been put forth to understand GPCR:PDZ protein interactions and their potential as drug targets^7–11^. For example, pharmacological disruption of the nNOS:NOS1AP:PSD95:NMDAR protein complex provides an alternative approach to NMDAR antagonists for treating neuropathic pain^12–14^ and neuronal excitotoxicity^15^, demonstrating the therapeutic potential of targeting PDZ protein interactions to selectively modulate membrane protein function.

Of the three α_1_-AR GPCR subtypes (α_1A_, α_1B_, α_1D_) that respond to the endogenous catecholamines epinephrine and norepinephrine, only the α_1D_-AR subtype contains a C-terminal Type I PDZ ligand. Yeast 2-hybrid^16^ and tandem-affinity purification/mass spectrometry^17^ screens initially revealed the α_1D_-AR PDZ ligand interacts with the syntrophin family of PDZ domain containing proteins. Syntrophins enhance α_1D_-AR function via recruiting the Dystrophin Associated Protein Complex (DAPC) and signaling effectors, α-catulin, liprin and phospholipase-Cβ^18^. Improved proteomic analyses subsequently revealed that, in addition to syntrophins, α_1D_-ARs also interact with the multi-PDZ domain containing protein scribble (SCRIB); and that α_1D_-ARs are expressed as modular homodimers, with one α_1D_-AR protomer bound to SCRIB, the other to syntrophin, in all human cell lines examined to date^19^. Strikingly, the α_1D_-AR:SCRIB:syntrophin complex is highly unique – no other GPCRs containing C-terminal Type I PDZ ligands have been shown to interact with both SCRIB and syntrophins^20^. Without significant expression of necessary PDZ proteins, α_1D_-ARs are retained intracellularly and produce weak functional responses^21–23^, suggesting this protein:protein interaction site has the potential for pharmacological modulation. Indeed, numerous diseases are associated with aberrant α_1_-AR function, including hypertension^24^, benign prostate hypertrophy^25^, bladder obstruction^26^, schizophrenia^27^, and post-traumatic stress disorder^28,29^. Unfortunately, deleterious side effects (i.e. orthostatic hypotension, reflex tachycardia) are frequently observed with chronic use of non-selective α_1_-AR antagonists. For example, the doxazosin portion of the ALLHAT anti-hypertensive study was prematurely halted due to increased morbidity^30^. Thus, selectively targeting the α_1D_-AR:SCRIB:syntrophin complex may provide therapeutic benefit, minus the toxicities associated with non-selective α_1_-AR ligands.

Herein, we employed a combination of biophysical, biochemical and cell-based approaches to acquire structural insights into the α_1D_-AR:PDZ protein complex. Together, our data implicate SCRIB PDZ domains 1 and 4 as the primary anchor sites for the α_1D_-AR. We further highlight differences in α_1D_-AR:PDZ1 versus α_1D_-AR:PDZ4 interactions by identifying unique residues in PDZ4 that are critical for α_1D_-AR binding.

## Results and Discussion

### α_1D_-AR preferentially binds SCRIB PDZ domains 1 and 4

**Figure 1:**
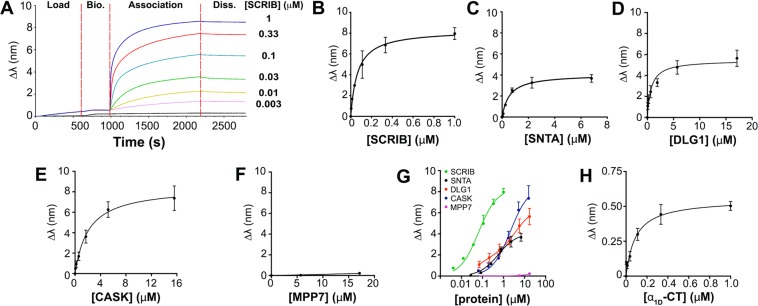
*In situ* affinity determination of α_1D_-adrenergic receptor C-terminal PDZ ligand:PDZ protein interactions. (**A**) Real-time biolayer interferometry (BLI) association/dissociation curve measuring binding of α_1D_ C-terminus (α_1D_-CT) to purified scribble (SCRIB). Biotin-labeled α_1D_-CT was immobilized to streptavidin probes. Indicated concentrations of SCRIB were used as analytes. (Bio. = Biocytin, Diss. = Dissociation). (**B**–**F**) Quantified BLI binding data for biotin labeled α_1D_-CT binding to (**B**) SCRIB, (**C**) α_1_-syntrophin (SNTA), (**D**) human discs large MAGUK scaffold protein 1 (DLG1), (**E**) calcium/calmodulin dependent serine protein kinase (CASK), and (**F**) membrane palmitoylated protein 7 (MPP7). (**G**) Comparative analysis of BLI concentration-response curves for α_1D_-CT:PDZ protein association binding. (**H**) Reverse BLI assay of purified α_1D_-CT (analyte) bound to immobilized biotin-labeled SCRIB (probe). Data are presented as mean ± SEM, n = 3.

We previously discovered the α_1D_-AR interacts with multiple PDZ proteins with cell-type specificity: scribble (SCRIB), α_1_-syntrophin (SNTA), human discs large MAGUK scaffold protein 1 (DLG1), and calcium/calmodulin-dependent serine protein kinase (CASK)^19^. With the goal of elucidating the molecular architecture of this unique, modular GPCR:PDZ protein complex, we employed BioLayer Interferometry (BLI) to quantify equilibrium dissociation constants (K_D_) for α_1D_-AR PDZ ligand:PDZ protein interactions. cDNAs encoding for the PDZ domains of these proteins were subcloned into a modified pGEX vector (pCOOL), expressed in *E. coli* and purified. Immobilized biotin-labeled peptides containing the distal 20 amino acids of α_1D_-AR (α_1D_-CT) were incubated with purified PDZ proteins and subjected to BLI analysis (Fig. 1A). We first compared α_1D_-CT binding to SCRIB and α_1_-syntrophin (SNTA), as α_1D_-ARs were found to interact with both PDZ proteins in all human cell lines examined^19^. Remarkably, α_1D_-CT bound SCRIB (K_D_ = 70 ± 20 nM; Fig. 1B) with ~8 higher affinity than SNTA (K_D_ = 0.56 ± 0.14 μM; Fig. 1C). DLG1 (K_D_ = 0.79 ± 0.21 μM; Fig. 1D) and CASK (K_D_ = 1.15 ± 0.21 μM; Fig. 1E), similar to SNTA, bind α_1D_-CT with lower affinity than SCRIB. MPP7, a known interactor of DLG1 and CASK^31^, displayed negligible α_1D_-CT binding (Fig. 1F). The combined rank order of affinity for α_1D_-CT interactions with known PDZ proteins is SCRIB» > SNTA > DLG1 > CASK» > MPP7 (Fig. 1G). α_1D_-CT:SCRIB binding affinity was validated by performing reverse BLI on GST-SCRIB probes incubated in serial dilutions of biotinylated α_1D_-CT (K_D_ = 76 ± 20 nM; Fig. 1H).

**Figure 2:**
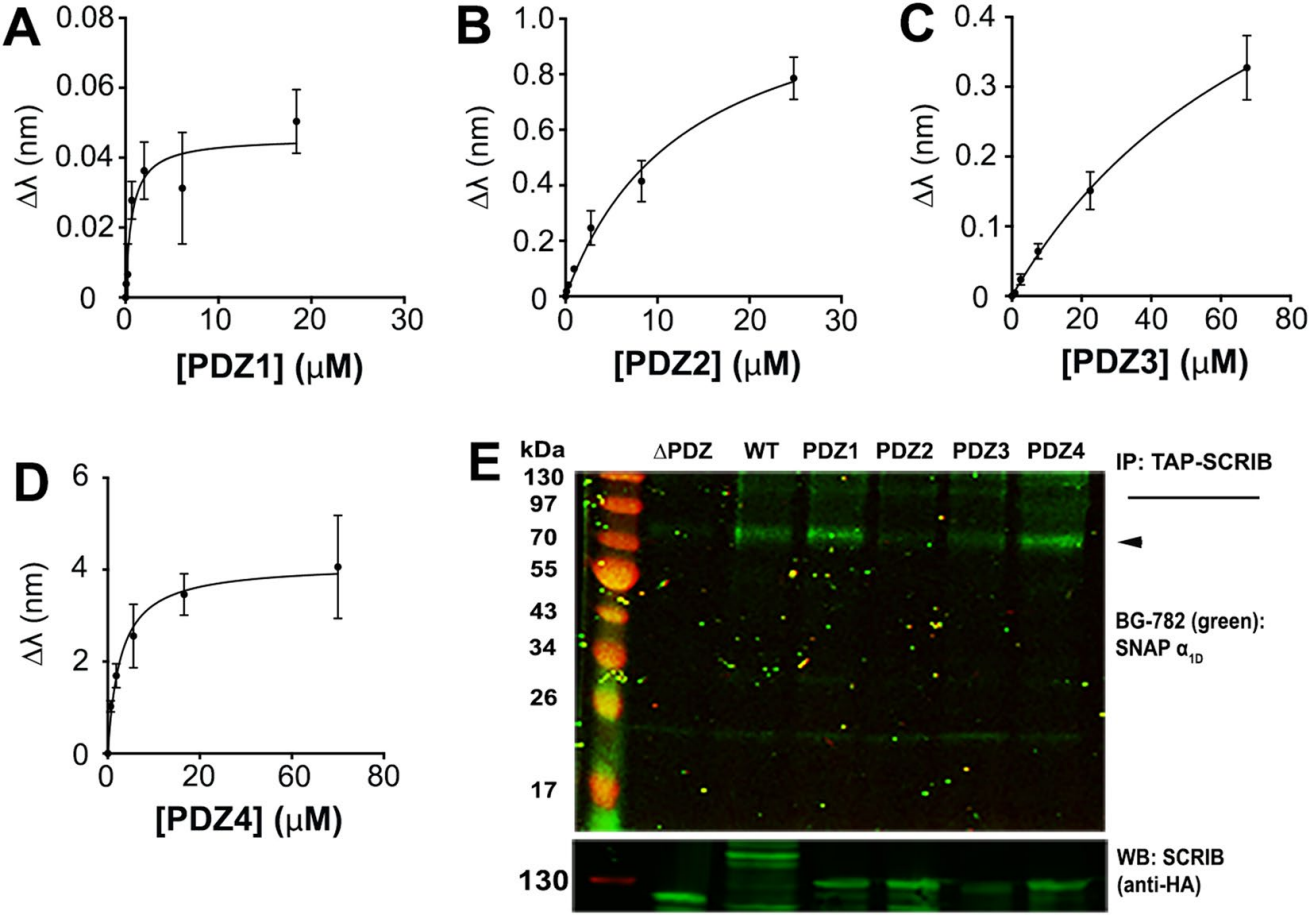
*In situ* and *in vitro* analysis of α_1D_-adrenergic receptor C-terminal PDZ ligand:SCRIB single PDZ domain interactions. (**A–D**) Biolayer interferometry (BLI) analyses of immobilized biotin-labeled α_1D_-CT binding to (**A**) SCRIB PDZ domain 1 (PDZ1), (**B**) SCRIB PDZ domain 2 (PDZ2), (**C**) SCRIB PDZ domain 3 (PDZ3) and (**D**) SCRIB PDZ domain 4. BLI data are presented as mean ± SEM, n = 3. (**E**) *Top panel*, PAGE NIR of BG-782 labeled SNAP-α_1D_-AR co-immunoprecipitated with TAP-SCRIB containing all 4 PDZ domains (WT), PDZ domain 1 (PDZ1), 2 (PDZ2), 3 (PDZ3) or 4 (PDZ4), or no PDZ domains (ΔPDZ) from HEK293 cell lysates. *Bottom panel*, Anti-HA western blot of upper gel for listed TAP-SCRIB constructs. ◄ indicates SNAP-α_1D_-AR monomer band.

A defining structural characteristic of SCRIB includes the presence of four clustered PDZ domains in the C-terminal portion of the polypeptide. Thus, we questioned if α_1D_-CT selectively associates with targeted PDZ domains on SCRIB. Individual PDZ domains were purified as GST-fusion proteins from *E. coli* and subjected to BLI analysis. SCRIB PDZ1 (K_D_ = 1.93 ± 0.49 μM; Fig. 2A) and SCRIB PDZ4 (K_D_ = 1.14 ± 0.23 μM; Fig. 2D) bind α_1D_-CT with the highest affinity, followed by SCRIB PDZ2 (K_D_ = 14.9 ± 5.44 μM; Fig. 2B) and SCRIB PDZ3 (K_D_ = 44.16 ± 13.52 μM; Fig. 2C).

Next, SCRIB containing all 4 PDZ domains (WT), SCRIB mutants containing single-PDZ domains (PDZ1, PDZ2, PDZ3, PDZ4), and SCRIB lacking all 4 PDZ domains (ΔPDZ) were subcloned into the pGlue vector to add N-terminal tandem affinity purification (TAP) epitope tags. HEK293 cells were transfected with TAP-SCRIB constructs and cell lysates were subjected to immunoblotting to verify expression (Suppl. Fig. 1). Next, constructs were transiently transfected into HEK293 cells stably expressing SNAP-α_1D_-AR. Cell lysates were affinity purified with streptavidin beads. Samples were labeled with BG-782 to detect SNAP-α_1D_-AR and imaged with PAGE NIR. As shown in Fig. 2E, SNAP-α_1D_-AR co-immunoprecipitated robustly with SCRIB WT, PDZ1, PDZ4, and to a lesser extent, with PDZ3. As expected SCRIB ΔPDZ produced no significant SNAP-α_1D_-AR binding. Thus, *in vitro* analysis of α_1D_-AR:SCRIB interactions concurs with prior *in situ* BLI results.

Taken together, these data implicate SCRIB PDZ1 and PDZ4 as the central scaffolds of the α_1D_-AR complex. Based on our discovery that CASK and DLG1 bind with relatively low affinity to the α_1D_-AR PDZ ligand, and that previous studies have reported SCRIB can interact with additional PDZ proteins (reviewed in^32^), we suspect CASK and DLG1 are recruited to the α_1D_-AR complex indirectly by SCRIB. For example, DLG1 can be indirectly recruited to SCRIB via GUKH, which interacts with SCRIB PDZ2 in *Drosophila* synaptic boutons^33^, or LGL − a known interactor with both DLG1 and SCRIB^34,35^. Additionally, DLG1, CASK, and LIN-7A are expressed as a tripartite complex *in vitro* and *in vivo*^36–38^, suggesting DLG1 may be recruiting CASK and LIN-7A to the α_1D_-AR complex via indirect interactions with SCRIB.

### α_1D_-CT:SCRIB binding is co-operative

**Figure 3:**
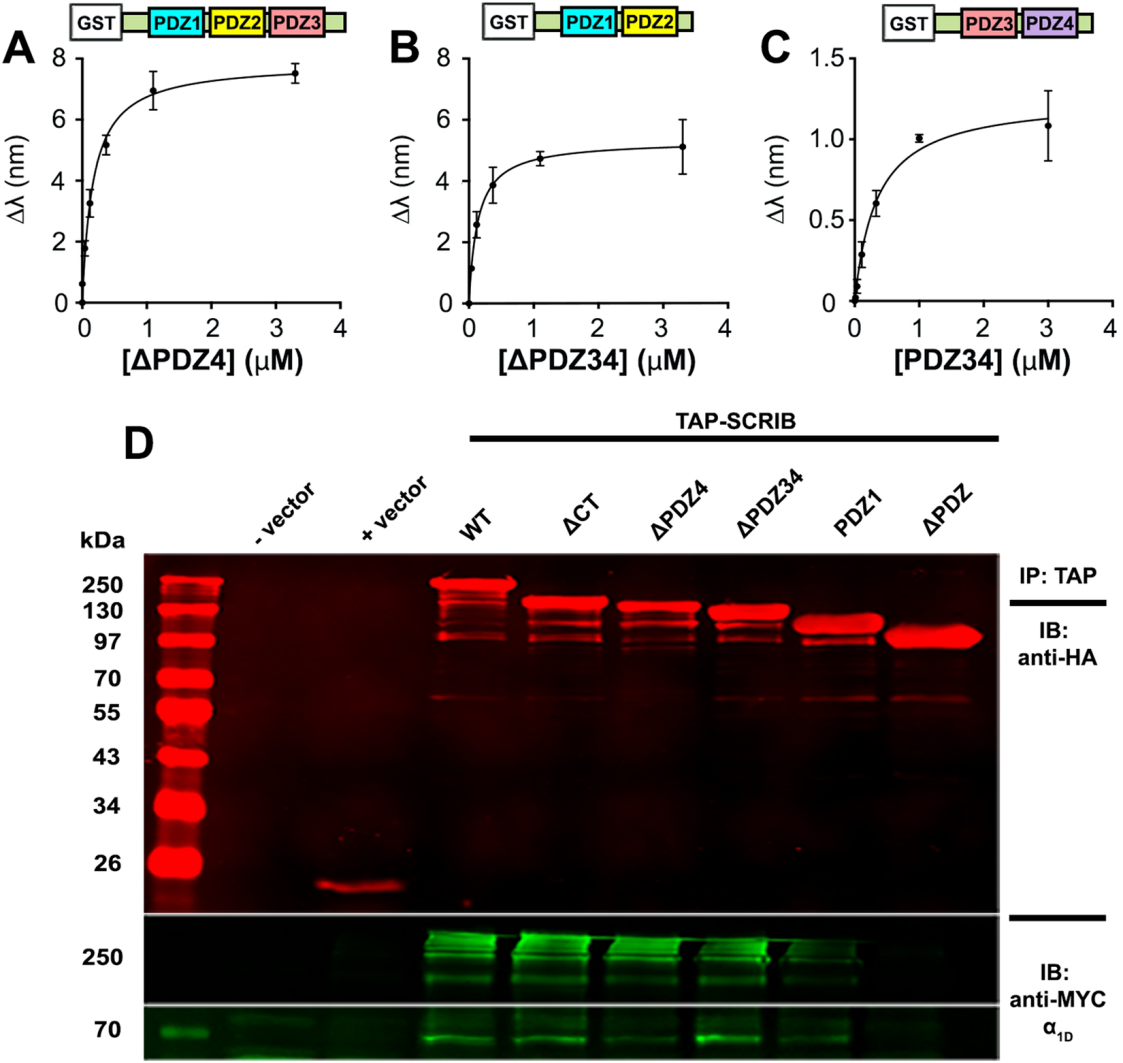
*In situ* and *in vitro* analysis of α_1D_-adrenergic receptor C-terminal PDZ ligand:SCRIB truncation mutant interactions. (**A–C**). Biolayer interferometry (BLI) analyses of α_1D_-CT binding to SCRIB ΔPDZ4 (**A**), SCRIB ΔPDZ34 (**B**) and SCRIB PDZ34 (**C**). BLI data are presented as mean ± SEM, n = 3. (**D**) Co-immunoprecipitation of myc-α_1D_-AR with transfection vehicle (− vector), empty pGlue vector (+vector), TAP-SCRIB containing all 4 PDZ domains (WT), or sequentially truncated at the C-terminus (CT), PDZ domain 4 (ΔPDZ4), PDZ domain 3 (ΔPDZ34), PDZ domain 2 (PDZ1) or PDZ domain 1 (ΔPDZ) from HEK293 cell lysates. Shown are western blots of TAP-SCRIB constructs (*top panel*), myc-α_1D_-AR multimers (*middle panel*) and monomers (*bottom* panel). For full blots reference Supplemental Fig. 2.

A key finding from BLI studies was the notable difference in α_1D_-CT binding affinity for SCRIB containing all 4 PDZ domains (70 nM) relative to each individual SCRIB PDZ domain (1.14–44.16 μM). The divergent α_1D_-CT:SCRIB binding affinities are suggestive of a co-operative binding mechanism, in that the binding of a single α_1D_-CT PDZ ligand to SCRIB enhances the affinity of subsequent intramolecular α_1D_-CT:SCRIB PDZ binding events. We tested this model by quantifying the affinity of SCRIB C-terminal truncation mutants missing PDZ4 (ΔPDZ4) or PDZ3 and PDZ4 (ΔPDZ34) with BLI. α_1D_-CT bound SCRIB ΔPDZ4 (K_D_ = 0.14 ± 0.02 μM; Fig. 3A) and SCRIB ΔPDZ34 (K_D_ = 0.16 ± 0.01 μM; Fig. 3B) with ~2x lower affinity than SCRIB WT (not significant, One-way ANOVA with Tukey’s post-hoc test), but ~6x higher affinity than SCRIB PDZ1 (p = 0.001, One-way ANOVA with Tukey’s post-hoc test) or PDZ4 alone (p = 0.07, One-way ANOVA with Tukey’s post-hoc test). In the reverse experiment, α_1D_-CT bound SCRIB PDZ3 and PDZ4 (PDZ34) with substantially lower affinity (K_D_ = 0.34 ± 0.09 μM; Fig. 3C; not significant, One-way ANOVA with Tukey’s post-hoc test) than SCRIB WT, but greater than SCRIB PDZ4 (not significant, One-way ANOVA with Tukey’s post-hoc test). Thus, these findings are suggestive of a co-operative α_1D_-CT:SCRIB binding modality, and provide additional support for previous studies suggesting SCRIB PDZ34 forms a “supramodule” binding site for PDZ ligands^39^.

We then tested the ability of SCRIB truncation mutants to co-immunoprecipitate with full length α_1D_-AR in mammalian cell culture. TAP-SCRIB mutants were co-transfected with myc-α_1D_-AR into HEK293 cells, digitonin-solubilized as cell lysates, immunoprecipitated with streptavidin beads and probed for anti-HA (TAP-SCRIB; Fig. 3D, Suppl. Fig. 2, *top panel*) and anti-myc (α_1D_-AR; Fig. 3D, *lower panels*). As shown, successive C-terminal SCRIB deletions produced progressive decreases in α_1D_-AR monomer (Fig. 3D, *bottom panel*, 68 kDa) and multimer (Fig. 3D, *middle panel*, ~250 kDa) signal, whereas SCRIB ΔPDZ produced no detectable α_1D_-AR interaction. Of note, the most dramatic decrease in α_1D_-AR binding was observed with SCRIB containing only PDZ1 (Fig. 3D, lane PDZ1).

**Figure 4:**
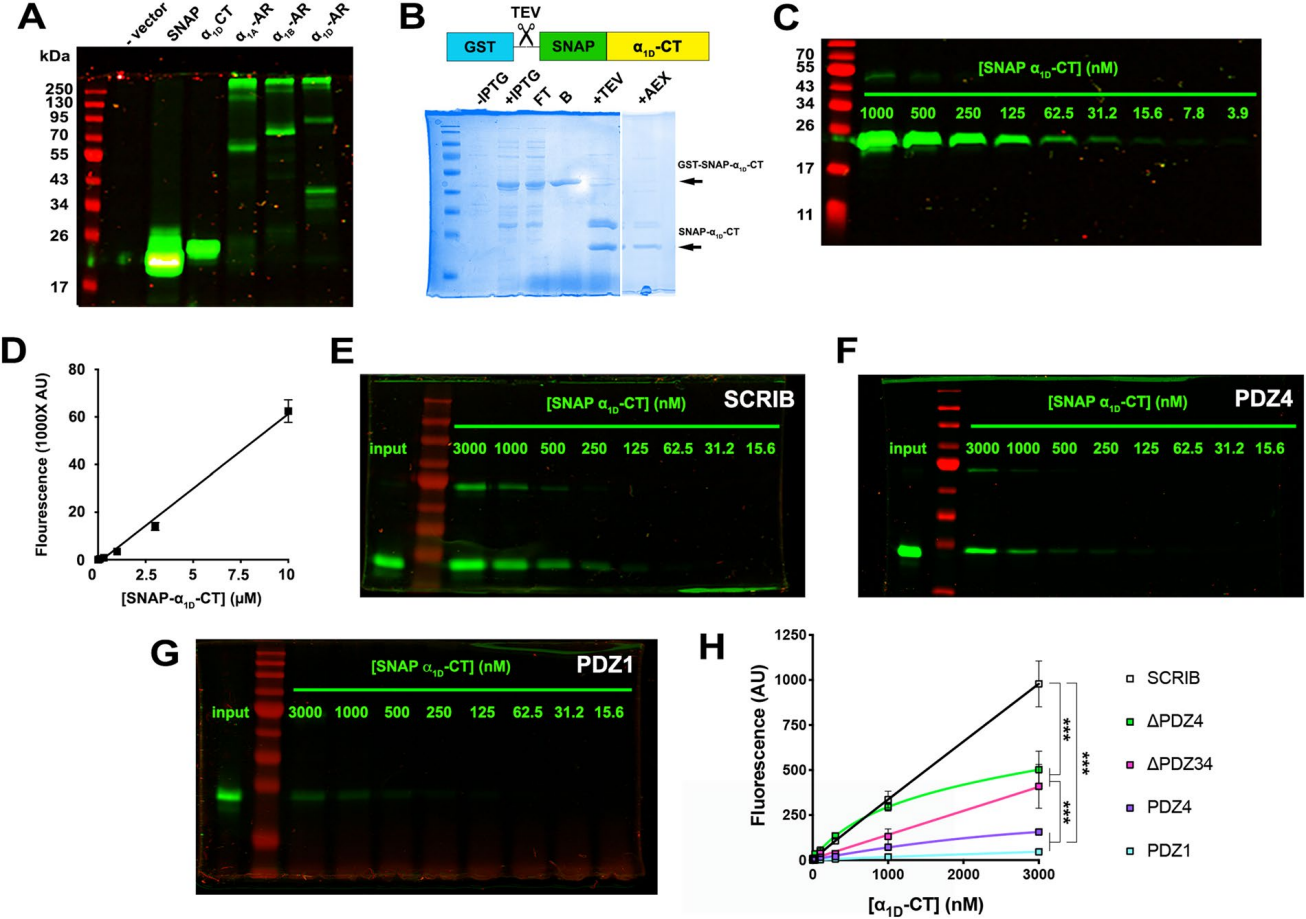
SNAP-α_1D_-adrenergic receptor C-terminal PDZ ligand:GST-SCRIB pulldown assays indicate a co-operative binding model. (**A**) PAGE NIR of HEK293 cell lysates transfected with vehicle alone (− vector), pSNAP vector (SNAP), N-terminal SNAP-tagged α_1D_-AR C-terminus (α_1D_-CT), α_1A_, α_1B_ and α_1D_-AR. (**B**) Coomassie stain of GST-SNAP-α_1D_-CT purification ± IPTG induction, unbound (FT), bound to beads (B), following TEV cleavage (+TEV) and anion exchange chromatography (AEX). (**C**) PAGE NIR of purified SNAP-α_1D_-CT pre-labeled with BG-782. (**D**) SNAP-α_1D_-CT standard curve plotting concentration of BG-782 labeled SNAP-α_1D_-CT versus fluorescence quantified at λ = 800 nm. (**E–G**) Representative PAGE NIR gels of SNAP-α_1D_-CT pulldowns with GST-SCRIB (**E**), GST-PDZ4 (**F**), or GST-PDZ1 (**G**). (**H**) Concentration-response curves quantifying SNAP-α_1D_-CT bound to GST-SCRIB, SCRIB truncated before PDZ domain (ΔPDZ4), before PDZ domain 3 (ΔPDZ34), SCRIB PDZ1, or SCRIB PDZ4 (mean ± SEM, n = 3–4). ***p < 0.001, One-way ANOVA with Tukey’s post-hoc test.

Next, SNAP GST-tag pulldown assays were used to test the proposed co-operative model of α_1D_-AR:SCRIB binding. The experimental approach involved the creation of a novel reporter construct: a SNAP-epitope tag adjacent to the N-terminus of the distal 16 amino acids of the α_1D_-CT (SNAP-α_1D_-CT). PAGE near infrared (NIR) analysis of HEK293 cell lysates transfected with SNAP-α_1D_-CT displayed protein bands of expected size 21.7 kDa (Fig. 4A). Next, GST-SNAP-α_1D_-CT was expressed in and purified from *E. Coli*, then eluted via TEV cleavage (Fig. 4B). SNAP-α_1D_-CT was pre-labeled with 1 μM SNAP-substrate BG-782 and subjected to PAGE NIR/LICOR Odyssey NIR imaging (Fig. 4C) to generate a standard curve (Fig. 4D). Glutathione agarose beads were incubated with previously described GST-SCRIB constructs, mixed with serial dilutions of labeled SNAP-α_1D_-CT, eluted, and analyzed with PAGE NIR. 10 μM BG-782 pre-labeled SNAP-α_1D_-CT was included in each gel as a normalization control (Fig. 4E–G, denoted as INPUT). In accordance with previous BLI experiments, SCRIB WT bound SNAP-α_1D_-CT in a concentration-dependent manner (Fig. 4E), with higher avidity than PDZ4 (Fig. 4F; p < 0.0001, One-way ANOVA with Tukey’s post-hoc test) or PDZ1 (Fig. 4G; p < 0.0001, One-way ANOVA with Tukey’s post-hoc test). Interestingly, SCRIB ΔPDZ4 (~48% of SCRIB WT; p < 0.0001, One-way ANOVA with Tukey’s post-hoc test) and ΔPDZ34 (~34% of SCRIB WT; p < 0.0001, One-way ANOVA with Tukey’s post-hoc test) produced maximal SNAP-α_1D_-CT binding responses that were less than SCRIB WT, yet greater than single SCRIB PDZ domain constructs (<10% of SCRIB WT, Fig. 4H; p < 0.0001, One-way ANOVA with Tukey’s post-hoc test). Taken together, our findings support the model that multiple α_1D_-AR CT PDZ ligands bind a single molecule of SCRIB via a co-operative mechanism.

A similar model has been proposed for numerous proteins containing multiple PDZ domains^40–43^. For example, PDZ domains 1 and 2 of PSD-95 exhibit greater affinity for binding partners Kv1.4, NR2B, and CRIPT when expressed in tandem^40^. The PDZ domains of syntenin also work co-operatively to bind syndecan dimers – syntenin PDZ2:syndecan interaction is a pre-requisite for syntenin PDZ1:syndecan binding^41,42^. A recent study similarly found that PDZ domains 2 and 3 of PTPN13 show enhanced binding for the PDZ ligand of APC when expressed together compared to individual domain constructs^43^. These previously characterized interactions further support our findings that the α_1D_-CT:SCRIB interaction is co-operative.

### Structure-function analyses identify R1110^PDZ4^ as a selectivity determinant for α_1D_-CT binding

We next compared α_1D_-CT:SCRIB binding parameters to previously identified SCRIB PDZ1 and PDZ4 interactors. SCRIB PDZ1 interacts with >20 proteins^32^, whereas PDZ4 interacts with NOS1AP^44^, APC^45^, p22phox^46^, NMDA receptor subunits GLUN2A and GluN2B^47^, and DLC3^48^. Remarkably, α_1D_-CT has the highest reported affinity to date of all reported SCRIB PDZ4 interactors. For example, the PDZ ligand of p22phox binds SCRIB PDZ4 with K_D_ = 40 μM^46^, whereas the NMDA receptor PDZ ligands bind SCRIB PDZ4 with K_D_ > 150 μM^47^. Thus, targeting SCRIB PDZ4 may provide the highest opportunity to disrupt α_1D_-AR function without perturbing other SCRIB complexes.

**Figure 5:**
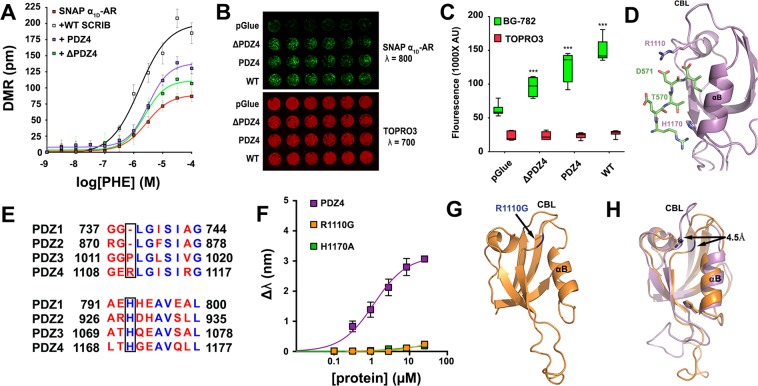
Structure-function analysis of the α_1D_-CT:SCRIB PDZ4 interaction. (**A**) Dynamic mass redistribution assays quantifying phenylephrine efficacy in HEK293 cells stably expressing SNAP-α_1D_-AR alone, or transfected with SCRIB WT, PDZ4, or SCRIB containing only PDZ domains 1, 2 and 3 (ΔPDZ4). Data are the mean of 12 replicates ± SEM. (**B**) Cell surface expression of SNAP-α_1D_-AR in HEK293 cells transfected with vector control (pGlue), ΔPDZ4, PDZ4, or SCRIB WT (*top panel, green*); nuclear stain TO-PRO-3 was used to normalize for cell number (*bottom panel, red)*. (**C**) Quantification of data from B (mean ± SEM, n = 3, 6 replicates; ***p < 0.001 from pGLUE, One-way ANOVA with Tukey’s post-hoc tests). (**D**) Molecular docking model of α_1D_-CT:SCRIB PDZ4 interaction (purple = PDZ4, green = α_1D_-CT, PDB ID = 4WYT used for model). (**E**) Sequence alignment of SCRIB PDZ domains (boxes indicate residues identified in **D**). (**F**) Biolayer interferometry (BLI) analysis of SCRIB mutations H1170A and R1110G on α_1D_-CT binding (mean ± SEM, n = 3). (**G**) X-ray crystallography structure of SCRIB PDZ4 R1110G (mutation highlighted in blue; PDB ID = 6EEY). (**H**) R1110G (orange) causes a 4.5 Å shift in carboxylate binding loop, as determined by superposition with WT PDZ4 (purple).

We first investigated the impact of SCRIB PDZ4 on α_1D_-AR functional responses. Label-free dynamic mass redistribution (DMR) signaling assays were performed using HEK293 cells stably expressing SNAP-α_1D_-AR alone or transiently co-expressing SCRIB WT, SCRIB PDZ4 or SCRIB ΔPDZ4. Concentration-response curves were generated for the selective α_1_-AR agonist phenylephrine to facilitate efficacy comparison between transfection conditions. As shown, phenylephrine efficacy was enhanced by all SCRIB constructs with rank order WT > PDZ4 > ΔPDZ4 > pGLUE vector control (Fig. 5A). Next, the ability of SCRIB mutants to promote α_1D_-AR plasma membrane trafficking were assessed using a 96-well plate near infrared imaging cell surface assay. The rank order of SCRIB constructs for promoting α_1D_-AR plasma membrane trafficking was WT > PDZ4 > ΔPDZ4 > pGlue (Fig. 5B,C). We have previously reported that α-syntrophin interacts with α_1D_-AR in the endoplasmic reticulum^49^, and that SCRIB and syntrophin co-localize and compete for the PDZ ligand of α_1D_-CT^19^. Therefore, we propose the α_1D_-AR:SCRIB interaction occurs in the endoplasmic reticulum to facilitate trafficking to the plasma membrane. However, further studies are warranted to determine the precise mechanism and machinery by which this complex formation is regulated.

Functional studies indicate targeting SCRIB PDZ4 alone may be a useful approach to modulate α_1D_-AR processes *in vivo*. However, this requires a thorough understanding of the structural determinants governing selectivity of the α_1D_-CT PDZ ligand for SCRIB PDZ4. Molecular docking that employed the solved crystal structure of SCRIB PDZ4 (PDB ID: 4WYT; refs.^39,50,51^) was used to predict α_1D_-CT:SCRIB PDZ4 interactions. Our model identified D571^α^^1D-AR^:R1110^PDZ4^ of the carboxylate binding loop and T570^α^^1D-AR^:H1170^PDZ4^ of α-helix B as possible α_1D_-CT PDZ ligand interaction sites within SCRIB PDZ4 (Fig. 5D). SCRIB PDZ domain sequence alignment revealed that R1110, but not H1170, is unique to PDZ4, suggesting that this residue may be responsible for the specificity of α_1D_-AR to PDZ4 (Fig. 5E). In support of our structural prediction, purified PDZ4 harboring either R1110G or H1170A mutations ablates α_1D_-CT binding (Fig. 5F).

Previous structural and biophysical studies have identified homologous histidine residues within Type I PDZ domains that control ligand specificity^52–55^. However, the structural role of R1110 is unknown. To resolve the mechanistic underpinnings of this interaction, the crystal structure of PDZ4 R1110G was solved to 1.15 Å resolution (Fig. 5G; Table 1; Suppl. Fig. S3). A superposition of the R1110G mutant with WT PDZ4 reveals a 4.5 Å shift of the carboxylate binding loop (Fig. 5H). We predict this shift creates steric hindrance that prevents the interaction between I572^α^^1D-AR^ and PDZ4. Previous studies have found PDZ ligand:PDZ domain interactions are dictated by interactions of the C-terminal residue of the PDZ ligand^52,55,56^. For example, *in situ* peptide library screens revealed 89% of peptides interacting with the PDZ domain of nNOS contain a C-terminal valine^56^. Thus, we propose that preventing I572^α^^1D-AR^ from interacting with PDZ4 is sufficient to inhibit α_1D_-CT binding.

**Table 1:** Data collection and refinement statistics for Scribble PDZ4 R1110G mutant (molecular replacement).

	SCRIB PDZ4 R1110G
**Data collection**	
Space group	P 1 21 1
Cell dimensions	
*a*, *b*, *c* (Å)	27.29, 40.24, 32.26
α, β, γ (°)	90, 97.85, 90
Resolution (Å)	31.96–1.145 (1.186–1.145)*
*R*_merge_	0.06674 (0.07563)*
*I*/σ*I*	12.82 (3.48)*
Completeness (%)	82.58 (3.40)*
Redundancy	4.4 (1.0)*
**Refinement**	
Resolution (Å)	31.96–1.145 (1.186–1.145)*
No. reflections	20613 (85)*
*R*_work_/*R*_free_	0.1571 (0.1385)/0.1818 (0.1574)*
No. atoms	1646
Protein	692
Ligand/ion	—
Water	131
*B*-factors	
Protein	7.36
Ligand/ion	—
Water	16.20
R.m.s. deviations	
Bond lengths (Å)	0.008
Bond angles (°)	1.35

*Values in parentheses are for highest-resolution shell.

**Figure 6:**
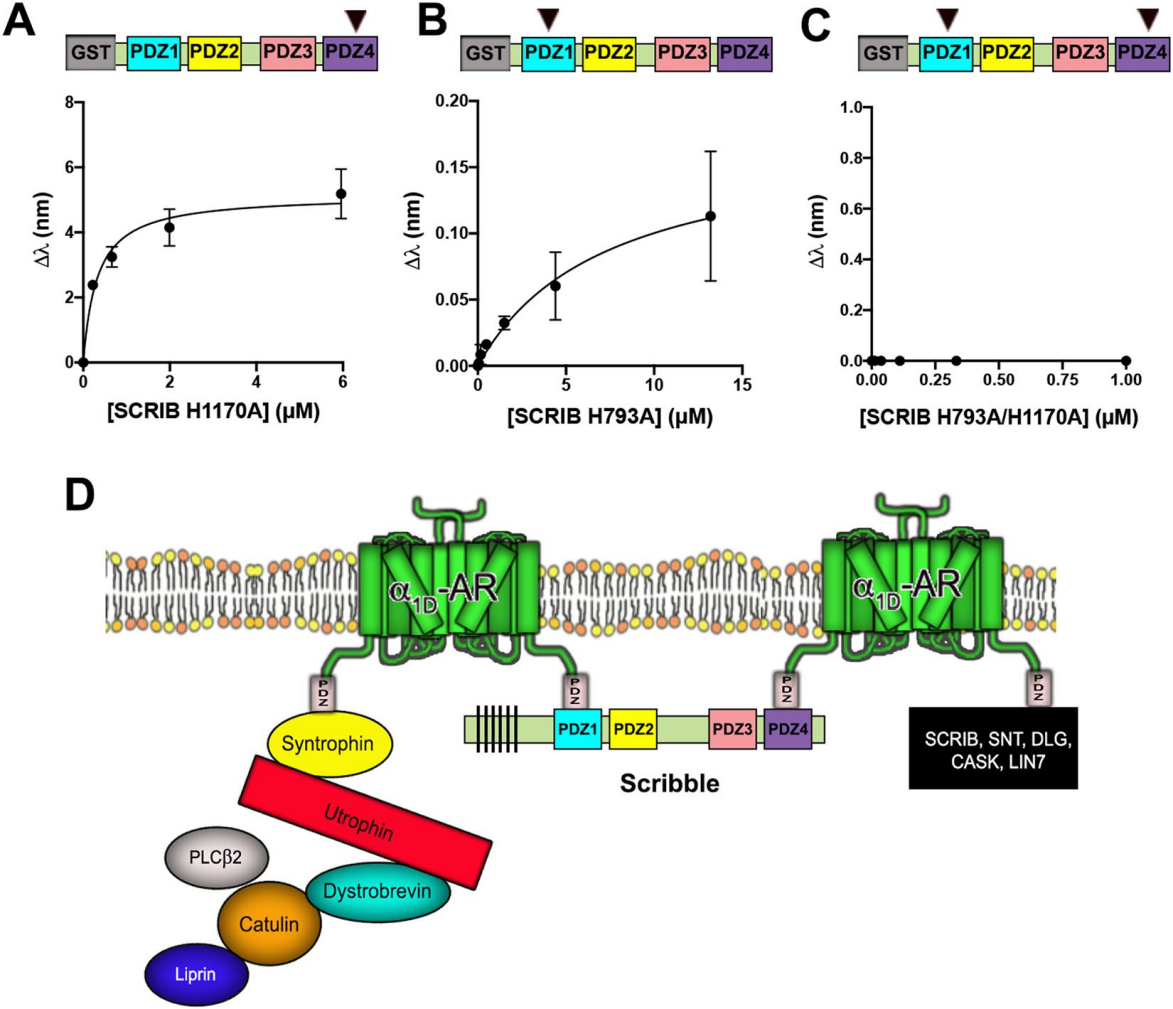
Biolayer interferometry analysis of α_1D_-adrenergic receptor C-terminal PDZ ligand:SCRIB H793A/H1170A interactions. Biolayer interferometry (BLI) was used to quantify α_1D_-CT binding to full length SCRIB containing point mutations H1170A (**A**), H793A (**B**), or both H793A and H1170A (**C**). ▼ indicate the SCRIB PDZ domain harboring the denoted H → A mutation. Data are presented as mean ± SEM, n = 3. (**D**) Hypothetical model of the α_1D_-AR:SCRIB:DAPC macromolecular complex in human cells.

Finally, we leveraged the information gathered from our structural studies to understand how mutations in either PDZ1 and/or PDZ4 affect the α_1D_-CT interaction in context of the core binding protein, SCRIB. This involved introducing H793A PDZ1 and H1170A PDZ4 mutations into GST-SCRIB (Fig. 6A, schematic) and subjecting to α_1D_-CT BLI analysis. As shown, SCRIB H1170A retains significant α_1D_-CT binding with affinity (K_D_ = 0.32 ± 0.08 μM; Fig. 6A) similar to the SCRIB PDZ34 construct (Fig. 3D). Mutating the equivalent amino acid in SCRIB PDZ1, H793A, produced a species that retains α_1D_-CT binding, with ~20x lower affinity (K_D_ = 7.34 ± 4.53 μM; Fig. 6B) than SCRIB PDZ4 H1170A. Strikingly, introducing both H793A and H1170A mutations into SCRIB abolished α_1D_-CT binding as measured by BLI (Fig. 6C).

## Conclusions

The present study strongly suggests that α_1D_-CT is capable of binding each SCRIB PDZ domain, but preferentially interacts with SCRIB PDZ1 and PDZ4. Also, the α_1D_-CT:SCRIB interaction appears to be co-operative, potentially driving multiple α_1D_-AR PDZ ligands to bind one molecule of SCRIB. We previously reported α_1D_-ARs can be expressed as modular homodimers in human cells, with one α_1D_-AR protomer bound to SCRIB, the other to syntrophin and the DAPC^19^. Based on the results of the present study, it is possible that α_1D_-AR homodimers interact simultaneously with both SCRIB PDZ1 and PDZ4, with at least one α_1D_-AR protomer interacting with the syntrophin:DAPC via the non-SCRIB bound PDZ ligand, and the other bound to a second syntrophin:DAPC module, or the DLG:CASK:LIN-7A tripartite complex (36–38; hypothetical schematic of α_1D_-AR:SCRIB:DAPC shown in Fig. 6D). Alternatively, an α_1D_-AR protomer may bind another SCRIB, anchoring interconnected α_1D_-AR:SCRIB:DAPC complexes at the plasma membrane in human cells. Finally, we demonstrate that SCRIB R1110^PDZ4^ serves as a unique α_1D_-CT interface site that could be targeted to modulate α_1D_-AR pharmacodynamics.

## Methods

### Plasmids and chemicals

Molecular cloning was performed using inFusion HD cloning technology (Clontech/Takara Biotech, Mountain View, CA). Constructs used for bacterial expression were sub-cloned into a modified pGEX vector to add GST-tags. For mammalian expression, constructs were inserted into pGLUE to add streptavidin binding protein/TEV/calmodulin binding protein tags; or pSNAPf to add SNAP-epitope tags; or pcDNA3.1 to fuse MYC tags. BG-782 SNAP substrate was from New England Biolabs (Ipswich, MA). PageRuler Prestained NIR Protein Ladder was used for all PAGE NIR (Thermo Fisher Scientific, Waltham, MA).

### Cell culture

Human embryonic kidney (HEK) 293 cells were grown in Dulbecco’s modified Eagle’s medium (DMEM) supplemented with 10% fetal bovine serum and 2 mM L-glutamine. Cells were transfected with 1 mg/ml polyethyleneimine (PEI) and used ~48 h post-transfection. For the development of the SNAP-α_1D_-AR stable cell line, G418 was added to the media 24 h post-transfection. [^3^H]-Prazosin saturation radioligand binding (data not shown) and PAGE NIR (described in 57) were used to verify SNAP-α_1D_-AR protein expression.

### Label-free DMR assays

DMR assays were performed in 384 well Corning Epic sensor microplates (Corning, Corning, NY) using the protocol described previously^57^. Data were analyzed with GraphPad Prism software (La Jolla, CA).

### Recombinant protein expression and purification

Recombinant proteins were expressed in Rosetta™ (DE3) competent cells (EMD Millipore, Burlington, MA) in Miller LB supplemented with 100 μg/mL Ampicillin and 34 μg/mL Chloramphenicol at 37 °C until an OD_600_ = 0.6–1.0 was reached; followed by induction with IPTG (1 mM) at 18 °C for 18 h. Cells were harvested by centrifugation and lysed (20 mM Tris-HCl pH 8.0, 200 mM NaCl, 5 mM DTT, Protease inhibitors). GST-tagged protein was immobilized on Pierce® glutathione agarose beads (Thermo Scientific, Waltham, MA) and washed (20 mM Tris-HCl pH 8.0 and 200 mM NaCl). Bound protein was eluted from the beads in wash buffer supplemented with 10 mM glutathione and concentration was determined using Bradford assay. Immobilized protein for crystallography was incubated with TEV at 4 °C for 18 h and subjected to size exclusion chromatography using a Superdex 75 Increase 10/300 GL (GE Healthcare, Chicago, IL) on an AKTA FPLC (GE Healthcare, Chicago, IL) in lysis buffer. The peak 215 nm fractions were collected. SDS-PAGE analysis was employed to determine purity, and protein was flash frozen and stored at −80 °C until needed.

### SNAP GST-pulldown assay

SNAP-α_1D_-C terminal domain (SNAP-α_1D_-CT) was created by subcloning cDNA encoding the distal 16 amino acids of the human α_1D_-C terminal domain into the 3′ MCS of pSNAP. SNAP and SNAP-α_1D_-CT were then subcloned into a modified pGEX vector to add N-terminal GST tags, expressed in, and purified from *E*. coli using the previously described method (Fig. 4B). Following TEV cleavage and ion exchange chromatography, SNAP-α_1D_-CT was reacted with BG-782 (1 μM) for 30 min @37 °C in the dark. Serial dilutions of BG-782:SNAP-α_1D_-CT were subjected to SDS-PAGE and near infrared fluorescence (NIR: λ = 800 nm) was quantified with the LI-COR Odyssey CLx (Fig. 4C; LI-COR, Lincoln, NE). Fluorescence intensity standard curves for SNAP-α_1D_-CT were generated to calculate protein concentrations (Fig. 4D). For GST-pulldown, 25 μL of 1 μM GST-tagged SCRIB proteins and 25 μL of BG-782:SNAP-α_1D_-CT were incubated with 25 μL of packed Pierce® glutathione agarose beads and rotated in the dark for 1 h at 4 °C. Samples were centrifuged @ 500 RPM at 4 °C for 5 min. Supernatant was discarded and beads were washed 3x (20 mM Tris-HCl pH 8.0, 200 mM NaCl, and 0.05% NP-40). Samples were boiled in SDS-sample buffer, and 10 μL aliquots were subjected to PAGE NIR.

### Affinity purification/Co-immunoprecipitation

TAP purification was performed using the protocol described previously^19,20^. 5 μL of 25 μM BG-782 was included in the 1^st^ overnight solubilization step with 0.5% digitonin to label SNAP-α_1D_-ARs. PAGE NIR was used to observe SNAP-α_1D_-AR protein levels. Gels were then transferred to nitrocellulose and blotted for anti-HA (#2367, Cell Signaling Technology, Danvers, MA) or anti-MYC (#9B11, Cell Signaling Technology, Danvers, MA), then anti-mouse Alexa-Fluor 2° antibodies in the 700–800 nm range (Invitrogen, Carlsbad, CA). Gels and blots were imaged with the LI-COR Odyssey CLx.

### Biolayer interferometry (BLI)

BLI was performed using the Octet Red 96 system (Pall Forte Bio, Fremont, CA). All steps were performed in 20 mM Tris-HCl pH 8.0, 200 mM NaCl, and 0.1% bovine serum albumin. 50 nM of biotin labeled peptide containing the last 20 amino acids of the α_1D_-CT (BioMatik, Cambridge, ON) was immobilized to streptavidin coated probes, followed by biocytin. The immobilized peptide was incubated in serial dilutions of target proteins until steady-state binding was reached. Biocytin was used to determine non-specific binding. For reverse BLI, GST-SCRIB was immobilized using anti-GST probes, and then incubated in serial dilutions of biotin labeled α_1D_-CT.

### Cell surface assay

HEK293 cell surface expression of SNAP-α_1D_-AR was quantified with cell impermeable SNAP-substrate BG-782 using the method described previously^57^. TO-PRO-3 nuclear stain was used to normalize samples according to cell number. Data were analyzed with GraphPad Prism software.

### X-ray crystallography

SCRIB PDZ4 R1110G was concentrated to 11 mg/mL in 20 mM Tris at pH 8.0, 200 mM NaCl, and 5 mM DTT and screened against crystallization conditions using a Mosquito Liquid Handler (TTP Labtech, Cambridge, MA). Final crystals were obtained in 21% PEG 3,350 and 0.25 M Ammonium Nitrate. Crystals were flash frozen in mother liquor supplemented with 15% glycerol. All diffraction data was collected at the Advanced Light Source at Berkeley on beam line 8.2.1, integrated with XDS^58^, and scaled with AIMLESS^59,60^. Phases were determined by molecular replacement using *Phaser*^61^ and SCRIB PDZ4 (39; PDB ID: 4WYT) as a search model. The *Phaser* solution was manually rebuilt over multiple cycles using Coot^62^ and refined using *PHENIX*^63^. All images were generated using the PyMOL Molecular Graphics System, Version 1.74 Schrödinger, LLC. Coordinate files have been deposited in the Protein Data Bank under the accession code 6EEY.

### Molecular docking

The distal 6 amino acids of the α_1D_-AR C-terminus, LRETDI, was modeled into the canonical βB and αB binding pocket of Scribble PDZ4^39^ using PyMOL and submitted to FlexPepDock server^50,51^. Models with scores greater than −131 were analyzed for hydrogen bonding (1.5–2.5Å) between peptide and PDZ4. Only interactions identified in greater than 5 models are reported.

## Supplementary information

Supplemental data
