# Supplementary material for: Scribble co-operatively binds multiple α1D-adrenergic receptor C-terminal PDZ ligands
Source: Sci Rep. 2019 Oct 1;9:14073. doi: 10.1038/s41598-019-50671-6 (PMC6773690; doi:10.1038/s41598-019-50671-6)
Supplement: Supplementary file 1 — Supplemental data [file 41598_2019_50671_MOESM1_ESM.pdf]

Scribble co-operatively binds multiple α_{1D}-adrenergic receptor C-terminal PDZ ligands

Eric M. Janezic[1§], Dorathy-Ann Harris[1], Diana Dinh[1], Kyung-Soon Lee[1], Aaron Stewart[1], Thomas R. Hinds[1], Peter L. Hsu[1,2], Ning Zheng[1,2], Chris Hague[1*]

[1]Department of Pharmacology, School of Medicine, University of Washington, 1959 NE Pacific Street, Seattle, WA 98195, USA
[2]Howard Hughes Medical Institute, University of Washington, Seattle, WA 98195, USA
[§]ORCID ID = 0000-0002-3177-889X

* To whom correspondence should be addressed: Chris Hague, Dept. of Pharmacology, University of Washington School of Medicine, 1959 Pacific Ave., Box 357280, Seattle, WA 98195; E-mail: chague@uw.edu; Tel. (206) 221-4612

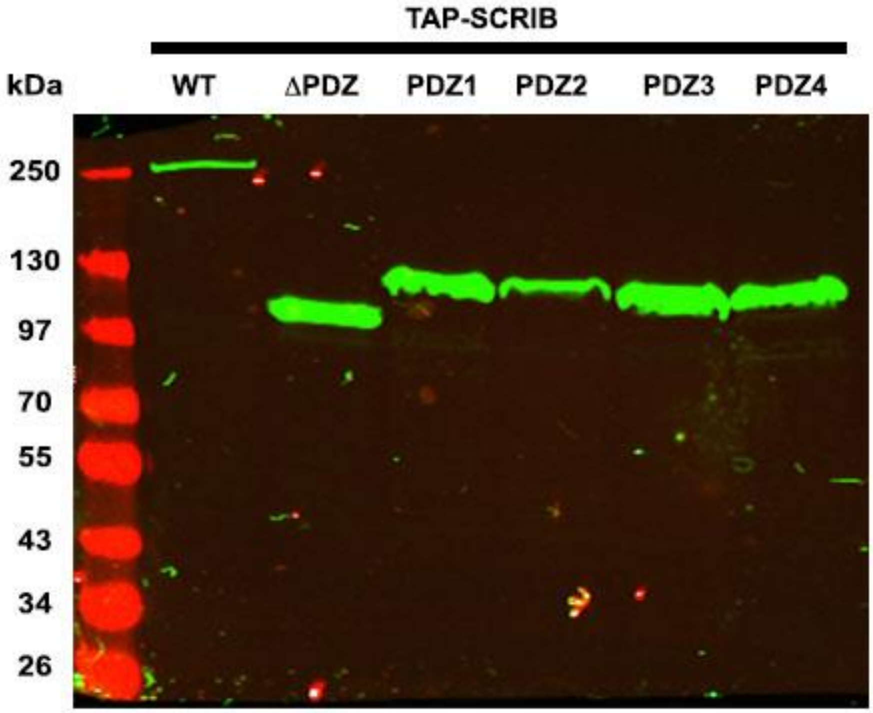

Supplement Figure 1. Western blot of TAP-SCRIB constructs probed with anti-HA antibody. Related to Figure 2D.

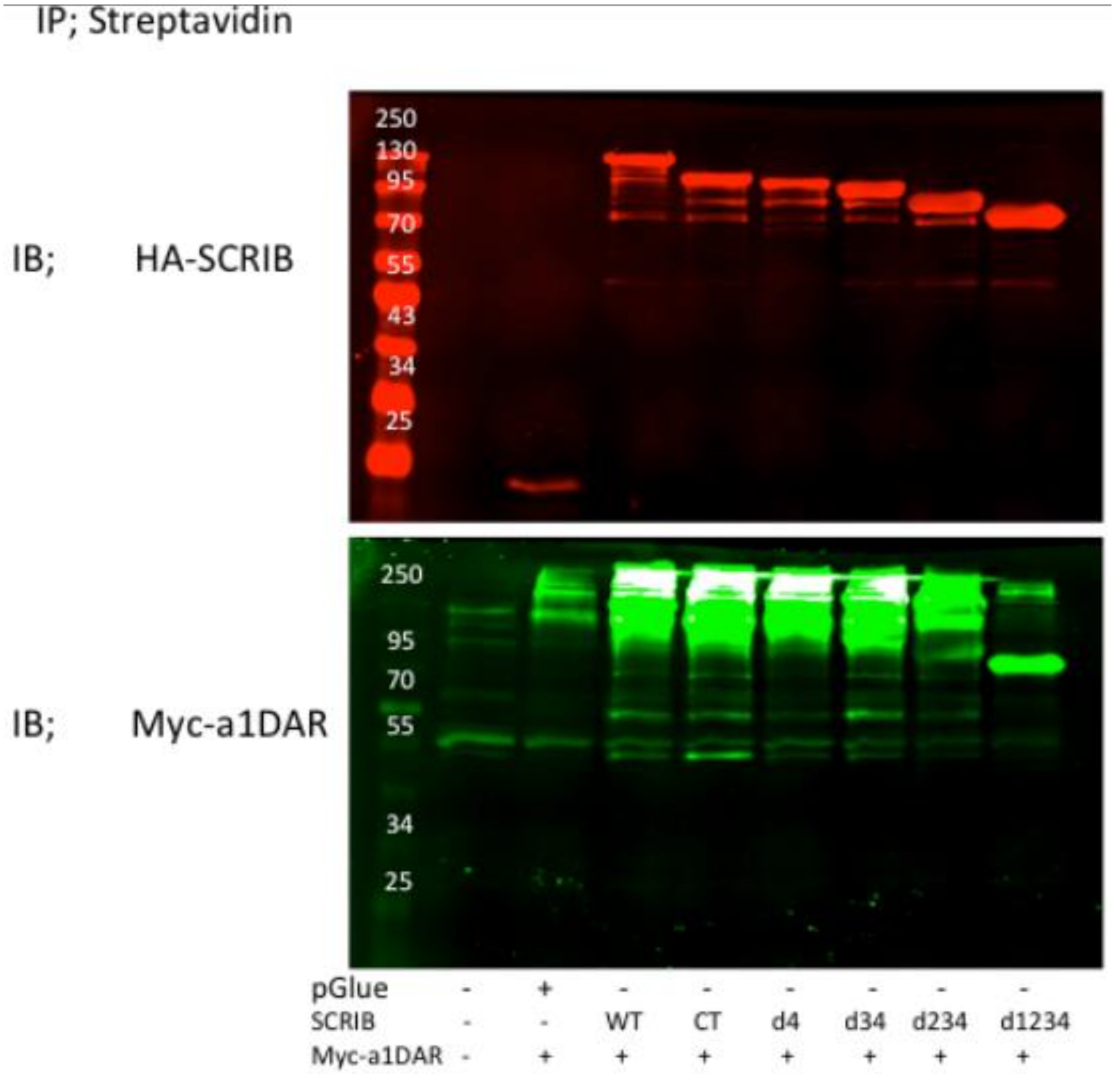

Supplemental Figure 2. Uncropped immunoblots from Figure 3D.

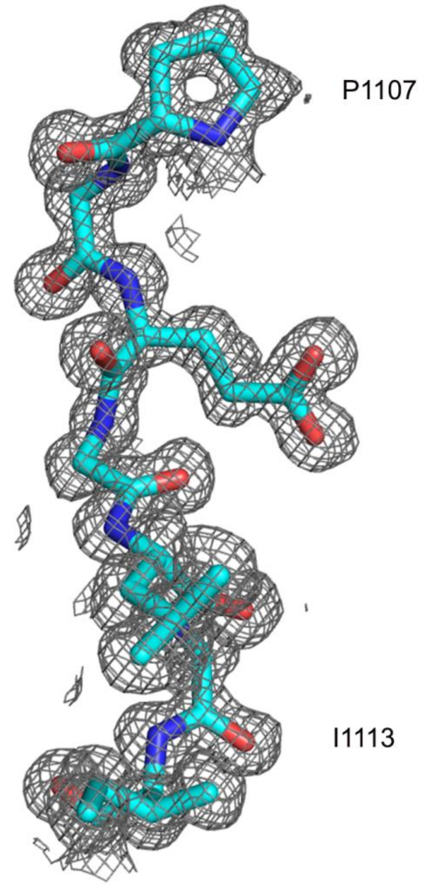

Supplemental Figure 3: Representative density map of PDZ4 R1110G superimposed with model.
